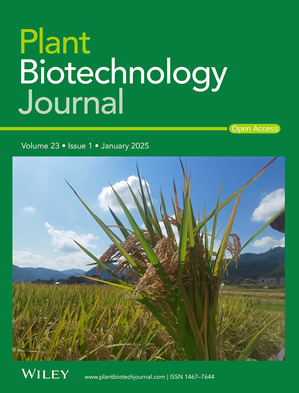# Issue Information

**DOI:** 10.1111/pbi.14387

**Published:** 2024-12-26

## Abstract

Front cover image:

A single mature plant of rice cultivar Pi‐4b with blast resistance. Cover illustration refers to the article published in this issue (Ma et al., pp. 250–267).